# Identification of Bufavirus-1 and Bufavirus-3 in Feces of Patients with Acute Diarrhea, China

**DOI:** 10.1038/srep13272

**Published:** 2015-08-19

**Authors:** Dou-Dou Huang, Wei Wang, Qing-Bin Lu, Jin Zhao, Chen-Tao Guo, Hong-Yu Wang, Xiao-Ai Zhang, Yi-Gang Tong, Wei Liu, Wu-Chun Cao

**Affiliations:** 1 Graduate School of Anhui Medical University, 230032, Hefei, P. R. China; 2 State Key Laboratory of Pathogen and Biosecurity, Beijing Institute of Microbiology and Epidemiology, 100071, Beijing, P. R. China; 3 School of Public Health, Peking University, 100191, Beijing, P. R. China

## Abstract

Bufavirus (BuV) is a newly discovered human parvovirus that has been detected in some countries. The current study was designed to understand the epidemic of BuV in China. Totally 1877 fecal specimens were collected from pediatric and adult patients with acute diarrhea in two large hospitals from 2010 to 2014. BuV was detected in 0.5% (9/1877) of the fecal samples by PCR and subsequent sequencing. The positive patients had a wide age range from 1 month through 60 years (median 24 years old) and 6 were male. A geographic specific pattern was obvious, with significantly higher frequency of BuV presented in Northern than in Southern China. Four BuV-1 and five BuV-3 were determined. Mixed-infections of BuV with sapovirus and novavirus were found in 2 cases, respectively. A temporal clustering was identified, with most positive detection focused in the cold weather. These findings have expanded the current knowledge on the geographic boundaries of BuV circulation.

Bufavirus (BuV) is a newly discovered human parvovirus that was firstly discovered from fecal specimens of a child with diarrhea in Burkina Faso in 2012^1^. The virus belongs to the species primate protoparvovirus 1 of the genus *Protoparvovirus*^2^. Sporadic human cases had been reported from multiple countries with various frequencies, including in Burkina Faso (4%,4/98), Tunisia (1.6%,1/63), Bhutan (0.8%,3/393), Finland (1.1%,7/629) and Netherlands (3.7%,1/27)^1^,^2^,^3^,^4^, but mostly from diarrhea patients. One child with acute flaccid paralysis also had BuV detected from fecal samples in Tunisia^1^, but the etiological causal relationship was undetermined at this moment.

BuV has a single-stranded DNA genome, encoding nonstructural protein 1 (NS1) and viral structural proteins 1 and 2 (VP1 and VP2). Based on VP1 and VP2 sequences, three genotypes, BuV1, BuV2 and BuV3, have been determined^1^,^2^. BuV1 and BuV2 were found in Burkina Faso, Netherlands, and Finland, while BuV3 was exclusively found in Bhutan, the only Asian country reporting BuV detection until recently. It is unknown whether BuV is circulating in China, where acute diarrhea remains to be severe contributor to morbidity in both children and adults. The objective of this study is to investigate the occurrence of BuV in diarrhea patients and to clarify its clinical significance and genetic characteristics in China.

## Methods

A retrospective study was performed in two large hospitals: the General Hospital of PLA (GHP) which is the largest general hospital in northern China serving the population from Beijing and neighboring areas, and he Children’s Hospital of Chongqing Medical University (CHCMU) which is the largest children’s hospital in southern China serving the pediatric patients from Chongqing and neighboring two provinces. All the recruited patients from the two hospitals were outpatients. As part of an ongoing project to identify viral etiology of diarrhea, patients with acute watery diarrhea attending infectious disease clinic in two hospitals were recruited since 2010. Patients who had any apparent clinical respiratory signs or symptoms were excluded. One stool sample was collected from each patient meeting the study criteria and immediately stored at −80 °C until laboratory tests. For comparison, stool samples that were collected from children without diarrhea who had sought medical care for other reasons in the same hospitals were used for the detection of BuV.

DNA/RNA was extracted from fecal samples by using QIAamp^®^ MinElute Virus Spin Kits (QIAGEN, Hilden, Germany) according to the manufacturer’s instructions. The presence of BuV was determined by applying real-time PCR as previously described^3^. The analytical sensitivity of the RT-qPCR assay was determined to be 5–10 copies per reaction. The positive samples were further subjected to nested PCR targeting the NS1 region^1^. For whole genome sequencing of positive samples, primers were constructed from consensus regions of the BuV whole-genome sequences (Supplemental materials.). PCR amplicons were directly sequenced by using Ion Torrent PGM sequencer (Thermo USA) according to the manufacturer’s instructions. Multiple sequence alignment was done by using Cluster W^5^ and the phylogenetic tree was constructed by MEGA 5.0^6^ using the neighbor-joining method. A bootstrap analysis of 1,000 replicates was done to determine the significance of branching. The samples had previously been tested for commonly seen enteric viruses, including norovirus, bocavirus, adenovirus, astrovirus and sapovirus by PCR, and for rotavirus by enzyme immunoassay using the previously described methods^7^,^8^,^9^,^10^. No tests for diarrheic bacteria were done on these samples. A standardized questionnaire was used to collect demographic information and clinical observations.

This study was performed with the approval of the Ethical Committees of Beijing Institute of Microbiology and Epidemiology and two hospitals. Written informed consents were obtained from all patients or the guardians of pediatric patients. The methods were carried out in accordance with the approved guidelines.

## Results

Totally 520 pediatric and adult patients recruited from GHP from 2010 to 2014 were tested for BuV. Their median age was 35 years old (range 1 month to 85 years old) and 310 were male. Totally 1357 pediatric patients recruited from CHCMU between 2010–2013 were tested for BuV, the median age was 10 months (range 1 day to 14 years old) and 828 were boys. Altogether 9 (0.5%) of the 1877 stool samples were positive for BuV DNA by both real-time PCR and PCR targeting NS1 segments. All nine positive detection were from patients recruited from GHP, with 2 (0.4%) found in 2010, 5 (1.0%) in 2011 and 2 (0.4%) in 2014. Totally 421 stool samples from children without diarrhea were detected (345 from CHCMU and 76 from GHP), from which no positive for BuV were found.

The patients with positive detection had a wide age range from 1 month through 60 years (median 24 years old) and 6 were male (Table 1). Higher frequency of BuV was found in GHP located in Northern China (1.7%) than in CHCMU located in Southern China (0%). A temporal clustering was identified, with all the positive detection focused in the cold weather (October, December, January and April), while not in summer (Table 1). This phenomenon was similar to that of the previous study^2^,^3^. Among all the seven enteric viruses detected, sapovirus and norovirus was co-detected in two patients with BuV infection, respectively. No else viruses were detected in the other 7 BuV positive patients. In addition to diarrhea, vomiting and abdominal pain were respectively observed in 1 adult. No fever or any other particular symptoms were reported from the positive patients.

**Figure 1:**
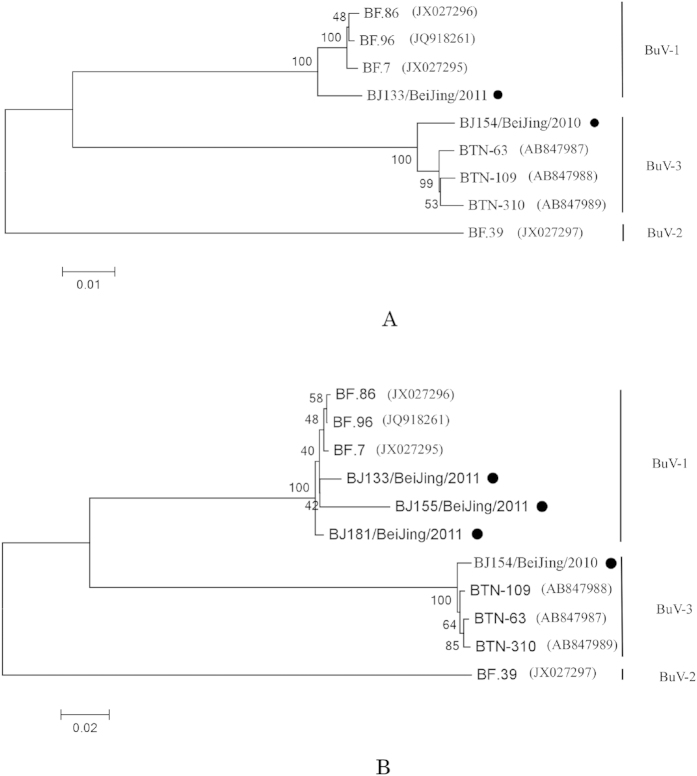
Phylogenetic trees were constructed based on the VP1 and the near-complete nucleotide sequences using maximum likelihood method with 1000 bootstrap by MEGA 5.0 for near-complete sequences (**A**) and VP1 (**B**). The strains in current study are labelled with black dots.

Two near-complete sequences, two partial VP1 and VP2 and five partial NS1 nucleotide sequences were used to perform Blastn analysis. Four sequences showed highest similarity with BuV-1 and 5 demonstrated high similarity with BuV-3 (Table 1). Phylogenetic analyses of VP1, VP2 and the near-complete sequences showed that F133 (Accession No KM580347), F155 (Accession No KM580349) and F181 (Accession No KM580351) were grouped into the Burkina Faso clade and F154 (Accession No KM580348) were grouped into Bhutan clade (Fig. 1).

## Discussion

This study is the first to document BuV in diarrhea patients in China, thus expanding the current knowledge on the geographic boundaries of BuV circulation^1^,^2^,^3^,^4^. The human BuV can infect individual of all age groups, and might possibly act as the etiological agent in the diarrhea patients. Spatially speaking, the virus was identified only in hospital of northern China, while not in that of southern China. This difference cannot be explained by age mismatch of patients from two hospitals, since the positive detection had been identified in all age groups. We therefore propose a geographic specific pattern for the BuV circulation, which, however needs to be corroborated by a more widely search based on large sample size in the future. The overall prevalence of BuV in China was comparable with that identified in Bhutan, yet lower than that from Africa. However, wider spread of the virus cannot be excluded, and surveillance in other regions is warranted. Different from previous studies, both BuV-1 and BuV-3 were determined, indicating a potential higher genetic complexity of Chinese strains. According to our results, higher frequency of BuV was identified in cold and dry seasons, which finding is consistent with the results from Bhutan, where the BuV- epidemic seasons were shown to be November, December and April. In the study in Finland, the BuV detection was similarly found in December, January and April. Compared with respiratory viruses, the seasonality of enteric viruses is less distinct, but a higher prevalence of viral over bacterial agents has been displayed from diarrhea patients in cold season, which pattern was displayed with a reverse trend in warm season, especially in Northern China^11^,^12^,^13^,^14^. The increased prevalence of BuV–associated diarrhea in cold season might be associated with the reduction of temperature or humidity.

It’also notable that single infection with BuV was identified in seven of the nine positive patients, indicating BuV might highly the etiological agents that caused the occurrence of acute diarrhea. However, no metagenomic studies have been done in the patients, therefore single-infection by BuV is only true relative to the list of viruses that are being tested. Although no BuV was detected from the non-diarrhea control, it’s yet unable to attain a causal link between the BuV and diarrhea. Evidence of seroconversion from convalescence samples is warranted to be sought and whether there is association between virus circulation and meteorological factors needs to be investigated.

## Additional Information

**How to cite this article**: Huang, D.-D. *et al.* Identification of Bufavirus-1 and Bufavirus-3 in Feces of Patients with Acute Diarrhea, China. *Sci. Rep.* **5**, 13272; doi: 10.1038/srep13272 (2015).

## Supplementary Material

Supplementary Information

## Figures and Tables

**Table 1:** Demographic information and tested enteric viruses in patients with bufavirus-positive diarrhea, China, 2010–2014*.

**Patient No.**	**Sample NO.**	**Age/sex**	**Other conditions than diarrhea**	**Disease onset date**	**Coinfected enteric virus**†	**BuV type**	**sequence length (bp)**	**GenBank Accession NO.**
1	F133	23Y/F	none	2011 Jan 16	−	BuV-1	4882	KM580347
2	F154	44Y/M	none	2010 Oct 29	sapovirus	BuV-3	4902	KM580348
3	F155	60Y/M	none	2011 Jan 17	−	BuV-1	3490	KM580349
4	F163	24Y/F	vomit	2010 Dec 1	−	BuV-1	1140	KM580350
5	F181	49Y/M	abdominal pain	2011 Jan 17	−	BuV-1	3612	KM580351
6	F189	50Y/M	none	2011 Jan 15	−	BuV-3	428	KM580352
7	F866	8Y/M	none	2011 Apr 2	−	BuV-3	413	KM580353
8	F2179	9Mo/F	none	2014 Apr 3	−	BuV-3	417	KM580354
9	F2180	1Mo/M	none	2014 Apr 6	norovirus	BuV-3	430	KM580355

^–^negative.

^*^Y, Years; Mo, month; M, male; F, female.

^†^Other detected enteric viruses included human bocavirus; human adenoviurs; norovirus; astrovirus; sapovirus; rotavirus.
