# Supplementary material for: Identification of Bufavirus-1 and Bufavirus-3 in Feces of Patients with Acute Diarrhea, China
Source: Sci Rep. 2015 Aug 19;5:13272. doi: 10.1038/srep13272 (PMC4541159; doi:10.1038/srep13272)
Supplement: Supplementary Information [file srep13272-s1.pdf]

Identification of Bufavirus-1 and Bufavirus-3 in Feces of Patients with Acute Diarrhea, China

Dou-Dou Huang[1,2#], Wei Wang[1,2#], Qing-Bin Lu[3], Jin Zhao[2], Chen-Tao Guo[1,2], Hong-Yu Wang[1,2], Xiao-Ai Zhang[2], Yi-Gang Tong[2], Wei Liu[1,2*] and Wu-Chun Cao[2*]

Affiliations:

[1] Graduate School of Anhui Medical University, 230032, Hefei, P. R. China;

[2] State Key Laboratory of Pathogen and Biosecurity, Beijing Institute of Microbiology and Epidemiology, 100071, Beijing, P. R. China;

[3] School of Public Health, Peking University, 100191, Beijing, P. R. China;

[#]These authors contributed equally to the paper.

*Correspondence authors:

Wu-Chun Cao M.D., Ph.D., State Key Laboratory of Pathogen and Biosecurity, Beijing Institute of Microbiology and Epidemiology, 20 Dong-Da Street, Fengtai District, Beijing 100071, P. R. China., Tel (+86)10-63896082, Fax (+86)10-63896082, E-mail: caowc@bmi.ac.cn

Wei Liu M.D., State Key Laboratory of Pathogen and Biosecurity, Beijing Institute of Microbiology and Epidemiology, 20 Dong-Da Street, Fengtai District, Beijing 100071, P. R. China., Tel (+86)10-63896082, Fax (+86)10-63896082, E-mail:lwbime@163.com

Supplemental Table Primer Sequences.

Primer_Name	Primer_sequence	Amplification_length
1-F	ATTTCATTCACCATGGCTCT	209bp
1-R	TGTTACTCACAGTTTGTTGG	
2-F	ATCACCTGGAAAGACTACAG	263bp
2-R	CACTGCAGACTAGTACATGG	
3-F	TTGGGAAAAGACACAGGCCT	194bp
3-R	TTAGCACATCAACCCAGTCA	
4-F	AGGCCTTACTACAACAAACC	293bp
4-R	GTTGTGGTTGGATTGTGGT	
5-F	CTTGGTTGCTAGGCACTGTC	281bp
5-R	TTTGACTAGGTCATAGGCAC	
6-F	CCTGCAGTGCTGTCTTAGAC	269bp
6-R	TAGCAGCCTGTGTTACCAAC	
7-F	TACTATGTGGGCCTGCAAGC	249bp
7-R	GGTTGTCATGACAACTGGAG	
8-F	GTGGCCAAGCAATCAGACTT	294bp
8-R	CCAATTTTCGCCCCATGTAG	
9-F	GGTCATACATAGACAGATGG	307bp
9-R	GTTGGCCATATCCTGTTGCC	
10-F	AGAAACCGAGCGAGCACTAG	308bp
10-R	TTTGCTTCTTGCTCTWGGAG	
11-F	CCTACTTCAACTGGAACAGC	269bp
11-R	GCTGAAGTCTTGATTGAAGG	
12-F	CCRCCTGGATACAACTACCT	284bp
12-R	GGCTGTATTCTGGTTCGGTG	
13-F	GCACTAGCTCCTGAACTGGC	312bp
13-R	GAGTGACAGACGATAGTAAC	
14-F	GGCACAGCACTGGYAATTAC	316bp
14-R	TGTCTAGTGTTTGGTCGAGG	
15-F	GATGAACCCAGCAGACTTCC	374bp
15-R	TGTTGTGGTTTCAATTGGAG	
16-F	CATGTCAACTTCTGGAACAC	426bp
16-R	CATGGTGCTTGGCTGAATGG	
17-F	CCACATAGGATACTCATGGC	346bp
17-R	CCCCATGGGTATTGTATTCC	
18-F	TCTGGACACAAGACTACCAC	281bp
18-R	CTTGGTAGTCTTAGTTTGCC	
19-F	AAACCTACAAACAGATGGAC	397bp
19-R	YATTGTGAATAGCAGCACCT	
20-F	ATGCCTGGAAGATGTATGCC	397bp
20-R	GTACTATCTGTTTGGTTGGT	
